# The brain limit

**DOI:** 10.7554/eLife.74096

**Published:** 2021-11-05

**Authors:** Alexander J Werth, Joseph E Corbett

**Affiliations:** 1 Department of Biology, Hampden-Sydney College Hampden-Sydney United States

**Keywords:** echolocation, biosonar, harbour porpoise, Blainville's beaked whale, predator-prey interactions, response latency, Other

## Abstract

How fast the brain and muscles can respond to information about prey location constrains visual and echolocating predators in similar ways.

**Related research article** Vance HM, Madsen PT, Aguilar de Soto N, Wisniewska DM, Ladegaard M, Hooker SK, Johnson M. 2021. Echolocating toothed whales use ultra-fast echo-kinetic responses to track evasive prey. *eLife* **10**:e68825. doi: 10.7554/eLife.68825

A small fish, darting about in the sea: tonight’s dinner for a savvy predator. If you, the reader, were the predator, you would only have your hands, teeth, and brain to catch the slippery creature that will respond to your approach with agile, evasive twists and turns (Moore and Biewener, 2015). Your eyes would track the fish through rapid, jerky movements that lock on to its new position 100–250 milliseconds after it has moved: this is the length of time it takes for your nervous system to process the information, and for your body’s muscles to respond (Erkelens, 2006).

Now imagine doing this in the dark, murky depths of the ocean – a challenging hunting ground for any optokinetic predator (that is, an animal that relies on its visual system to catch its prey). Toothed whales, such as dolphins and porpoises, have solved this quandary by evolving an echolocation system. Like bats, they emit a series of sonic clicks and then listen and analyze the echoes that bounce off the objects around them, including nearby meals. When the whales switch from slowly stalking to actively pursuing prey, they rapidly increase their click rate to get more information about their target’s location, creating a rapid train of sounds called a buzz (Madsen and Surlykke, 2013).

Until now, the echolocation of toothed whales was presumed to work on a synchronous ‘click-by-click’ basis, like human sonar systems: a new click would be produced as soon as the previous one had been received and processed (Au, 1993). However, given tooth whales’ rapid buzz rates, the animals would need to process information and respond to it 100 times faster than optokinetic predators (Kirchner and Thorpe, 2006). Is this the case, or do whales rely on a different strategy? Now, in eLife, Heather Vance from the University of St Andrews and colleagues based in the United Kingdom, Denmark, Spain and France report a new approach that sheds light on how toothed whales keep up with this rapid train of stimuli (Vance et al., 2021).

The team used suction cups to temporarily attach small electronic tags onto free-ranging beaked whales and harbor porpoises. The sensors recorded outgoing sonar clicks and returned echoes, as well as fine-scale data on the animals’ movement (such as their direction, speed and acceleration; Johnson and Tyack, 2003). Vance et al. then used this data to determine how close the predators were to their prey, how rapidly they sent and received clicks, and how they moved in response.

Results from the tags revealed that during the final moments of a pursuit, clicks are just two to three milliseconds apart – indicating that these rapid buzzes contain the equivalent of 500 clicks per second. Yet, it took the whales 100–200 milliseconds to physically respond to the echo, which is comparable to the delayed response time of optokinetic predators (Erkelens, 2006; Kirchner and Thorpe, 2006; Engbert et al., 2011). Experiments with captive harbor porpoises trained to respond to randomly moving target balls confirmed these findings. Overall, beaked whales’ reaction times were slightly longer than those of the smaller porpoises – suggesting that larger bodies respond more slowly. Nonetheless, both species shared strikingly similar response times given that they have evolved independently for the past 20 million years (Berta et al., 2014).

Overall, if whales processed sensory data on a click-by-click basis (as originally supposed), they would show much faster reaction times. These findings therefore refute the original ‘click-by-click’ claim whereby echolocators would wait to respond to an echo before releasing a new click. Instead, they suggest that both these ‘echokinetic’ animals (as named by Vance et al.) and optokinetic predators share a common neural mechanism that evolved in an earlier common ancestor – but when and in which species remains a mystery. In both groups, hunting responses are limited by the speed at which the brain can process information, and the muscles can respond (Figure 1). So, why do toothed whales produce clicks 100 times faster than they can process and respond to them?

**Figure 1.**
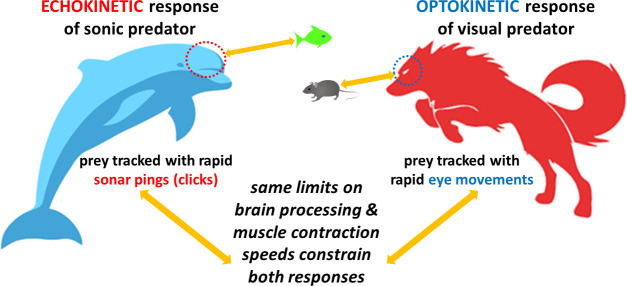
Comparing the response times of echolocating and visual predators. (Left) Toothed whales (such as dolphins and porpoises) are echokinetic predators that track prey by emitting sonar pings, or clicks, which they release faster as they get closer to their target: they then process the sensory information from the returning echo and move their bodies accordingly to hunt down the prey. (Right) Optokinetic predators (such as wolves) rely on their vision to hunt, rapidly moving their eyes to lock on to the prey’s position and track where it goes. Vance et al. found that echokinetic predators respond to the sensory input from the echo and coordinate their bodily motion at the same speeds that optokinetic predators use to track prey with eye movements. This suggests that the tracking abilities of both types of predators are limited by how fast their brains process sensory information and how quickly their muscles contract; it also indicates a shared evolutionary origin for the neuromuscular system that may underlie hunting in mammals.

Image credit: Alexander J Werth and Joseph E Corbett.

The answer likely lies in the fact that clicks are produced by the rapid vibration of nasal valves (or ‘phonic lips’) as air passes through them – similar to the squeaky sounds that emerge from air escaping from a balloon (Berta et al., 2014). How fast the clicks are created depends on the tension of the phonic lips and air pressure; it is likely that this anatomical process is not as finely controlled as the contraction of muscles involved in swimming and chasing prey. However, this creates a sensor equivalent to the fovea, an eye region densely packed with light-sensitive nerve cells that provides the sharpest vision. The densely packed (timewise) buzz of rapid clicks may create an equally sensitive ‘echolocatory fovea’ that allows the predator to integrate multiple echoes caused by the prey’s movements.

Toothed whales are not the only predators in the sea, and not all of them can perform echolocation. For example, seals use the whiskers on their snouts and surrounding their large eyes to sense the tiny water vibrations produced by moving prey (Dehnhardt et al., 1998). Yet, whether mammals use their eyes, whiskers or sonic pings, the results by Vance et al. suggest that they may all rely on roughly the same neurological processes to catch their dinner.
